# Telomere length in relation to fecundability and use of assisted reproductive technologies: the Norwegian Mother, Father, and Child Cohort Study

**DOI:** 10.21203/rs.3.rs-4430021/v1

**Published:** 2024-06-03

**Authors:** Karoline Hansen Skåra, Yunsung Lee, Astanand Jugessur, Håkon K. Gjessing, Abraham Aviv, Ben Brumpton, Øyvind Naess, Álvaro Hernáez, Hans Ivar Hanevik, Per Magnus, Maria C. Magnus

**Affiliations:** Norwegian Institute of Public Health; Norwegian Institute of Public Health; Norwegian Institute of Public Health; Norwegian Institute of Public Health; The State University of New Jersey; K.G. Jebsen Centre for Genetic Epidemiology, NTNU – Norwegian University of Science and Technology; University of Oslo; Norwegian Institute of Public Health; Norwegian Institute of Public Health; Norwegian Institute of Public Health; Norwegian Institute of Public Health

**Keywords:** Assisted reproductive technologies, fecundability, infertility, Mendelian randomisation, MoBa, MRBN, telomere length

## Abstract

In women, shorter telomeres have been reported to be associated with conditions such as endometriosis and polycystic ovary syndrome, whereas other studies have reported the opposite. In men, studies mostly report associations between shorter telomeres and sperm quality. To our knowledge, no studies have thus far investigated the associations between TL and fecundability or the use of ART.

This study is based on the Norwegian Mother, Father, and Child Cohort (MoBa) Study and uses data from the Medical Birth Registry of Norway (MBRN). We included women (24,645 with genotype data and 1,054 with TL measurements) and men (18,339 with genotype data and 965 with TL measurements) participating between 1998 and 2008. We investigated the associations between leukocyte TL and fecundability, infertility, and the use of ART. We also repeated the analyses using instrumental variables for TL, including genetic risk scores for TL and genetically predicted TL.

Approximately 11% of couples had experienced infertility and 4% had used ART. TL was not associated with fecundability among women (fecundability ratio [FR], 0.98; 95% confidence interval [CI], 0.92–1.04) or men (FR, 0.99; CI, 0.93–1.06), nor with infertility among women (odds ratio [OR], 1.03; CI, 0.85–1.24) or men (OR, 1.05; CI, 0.87–1.28). We observed an increased likelihood of using ART with increasing TL among men (OR, 1.22; CI, 1.03–1.46), but not among women (OR, 1.10; CI, 0.92–1.31). No significant associations were observed using the instrumental variables.

Our results indicate that TL is a poor biomarker of fecundability, infertility and use of ART in MoBa. Additional studies are required to replicate the association observed between TL and ART in men.

## Introduction

Ageing is related to increased prevalence of several diseases arising from an inevitable and irreversible decline in physiological function across multiple organ systems (1). It involves a gradual accumulation of molecular and cellular damage, including DNA mutations, oxidative stress, and telomere shortening (2). Telomeres are DNA-protein structures located at the ends of each chromosome. They consist of 5′-TTAGGG-3′ tandem repeats that serve as protective caps to prevent chromosomal degradation during DNA replication, thus maintaining genomic stability and preserving genetic information across cell divisions (3, 4). Telomere length (TL) is commonly measured in human blood lymphocytes (5). It is highly heritable across generations and gradually shortens with age, triggering cellular senescence or apoptosis upon reaching a critical threshold (4, 6, 7).

Fecundability, the probability of conceiving within a single menstrual cycle, declines with age (8–10). This decline in women is largely attributable to changes such as a decrease in the number and quality of oocytes and altered hormonal levels (8). While the impact of age on male infertility is less pronounced, there is evidence of a slight decline in semen quality (11). However, these factors only partly explain the decrease in fecundability with age in both sexes (12). It has been proposed that variations in TL could explain this decrease, and potentially act as a biomarker for low fecundability (13, 14).

Current evidence supports an association between TL and pseudo-measures of fecundability, such as premature ovarian failure, oocyte maturation, polycystic ovary syndrome (PCOS) and endometriosis (15, 16). However, studies investigating these associations have produced conflicting results (15, 16). For endometriosis, for instance, some studies report an association with longer TL (17, 18), but others with shorter TL (19, 20). Most studies on the relationship between TL and sperm quality in men suggest that shorter TL is associated with infertility-related sperm characteristics, although some studies also report associations with longer TL (15, 21). Generally, studies investigating the association between TL and reproductive potential generally involve modest sample sizes, ranging from approximately 30 to 1,200 participants (15). Crucially, none of the above studies have specifically addressed fecundability or the use of assisted reproductive technologies (ART). Additionally, previous studies have not considered the use of genetic risk scores (GRSs) for TL, which could offer insights into the role of unmeasured confounding factors (22, 23). The relationship between TL and fecundability among both women and men therefore remains unclear.

Given these important knowledge gaps, we aimed to investigate whether TL was associated with fecundability, infertility, or use of ART among women and men participating in the Norwegian Mother, Father, and Child Cohort Study (MoBa). We hypothesised that there would be an association between TL and fecundability, infertility, the use of ART among both women and men.

## Methods

### Study population

We studied participants in the Norwegian Mother, Father, and Child Cohort Study (MoBa), a population-based pregnancy cohort in which pregnant women and their partners were recruited around the 17th week of gestation between 1998 and 2008 (24, 25). Blood samples were collected at recruitment (26, 27). The majority of participants have been genotyped, quality-controlled as outlined in Corfield, Frei (28).

**Figure 1:**
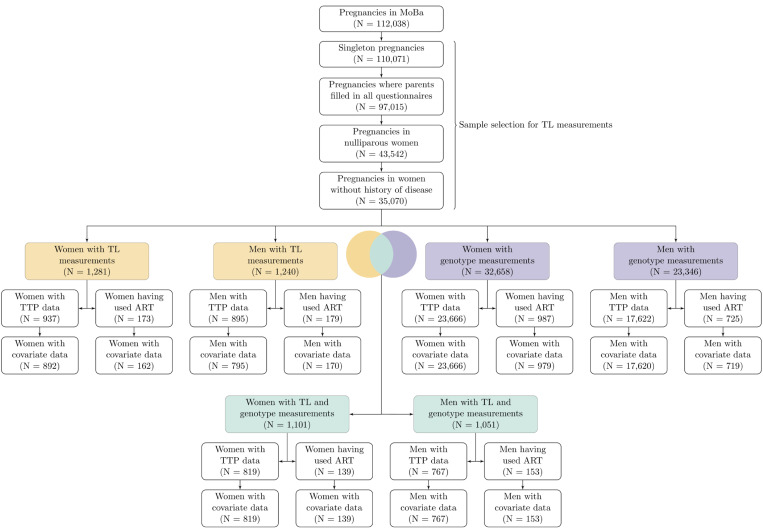
Flow chart of the study population. Detailing the participants for whom we had (i) only telomere length (TL) measurements (highlighted in yellow), (ii) only genotypes (in purple), and (iii) both TL measurements and genotypes (marked in green).

TL measurements were specifically conducted on a subset of nulliparous women and their partners who had singleton pregnancies, had completed the recruitment questionnaires administered to women and men, and had completed the questionnaire administered to women at 30 gestational weeks. Women with a history of cancer, diabetes, hypertension, preeclampsia, autoimmune disease, and intrauterine infections were not sampled for TL measurements, nor were women who gave birth to stillborn babies, babies with congenital malformations, or babies with small/large for gestational age (Fig. 1). For the present study, we only included women and their partners who reported their time to pregnancy (TTP) or had conceived using ART.

The Regional Committee for Medical and Health Research Ethics of South-East Norway (REK 2017/1362) approved this study. Our work is described according to the Strengthening the Reporting of Observational Studies in Epidemiology (STROBE) guidelines for reporting Mendelian randomisation and cohort studies. A written informed consent was obtained from all participants.

### Telomere measurements

Leukocyte TL has been measured in 1,597 women and 1,582 men. After applying the selection criteria mentioned above, pregnancies were randomly selected for the study and classified according to ART use. For studying ART, TL was only measured in women aged 30 years or above. In contrast, among women conceiving through sexual intercourse, TL was measured in women aged 18 years or above, with a targeted oversampling of women approximately 32 years of age (Supplementary Figure S1). The terminal restriction fragment (TRF) method was used to measure TL in leukocytes using Southern blot, as previously described (29). To adjust TL for age, we calculated residual TL by regressing TL against the age at which TL was measured, thus obtaining age-corrected TL (hereafter referred to as aTL; Supplementary Figure S2).

### Genetics of telomeres

To mitigate the impact of unmeasured confounding that often bias observational studies, and to enhance the statistical power of our study, we calculated GRSs as instrumental variables for TL based on the framework for one-sample Mendelian randomisation analyses (30). We identified independent single-nucleotide polymorphisms (SNPs) that were significantly associated with leukocyte TL (P < 5×10^−8^) based on the most recent genome-wide association study (GWAS) of TL by Codd, Wang (31). Of the 197 SNPs identified in Codd, Wang (31), only 120 were present in our MoBa genotype dataset. To handle the missing SNPs, we searched for substitute SNPs within a one-megabase range of the absent SNPs that were in strong linkage disequilibrium with the missing SNPs (R^2^ > 0.9), bringing our total number of SNPs to 144 for computing GRSs.

The GRSs for TL were calculated by summing the weighted risk alleles using effect sizes from Codd, Wang (31), applying the formula: $GRS=\sum_{i=1}^{m}\beta_{i}SNP_{i}$, where $\beta_{i}$ represents the effect of the *i*th SNP, and $SNP_{i}$ is the number of effect alleles for the *i*th SNP. We also used the GRSs to estimate genetically predicted aTL through two-stage least square (2SLS) regression, adjusting for the first ten ancestry-informative genetic principal components, as outlined by Burgess and Thompson (32).

### Fecundability, infertility, and use of assisted reproductive technologies

We used self-reported TTP as a measure of fecundability, defined as the probability of conceiving during a given menstrual cycle. Women indicated whether their pregnancy was planned and, if so, the time spent trying to conceive. For this variable, the options were “<1 month,” “1–2 months,” or “3 ≥ months”. We assigned a TTP value of 1, 2 and 3 months to these categories, respectively. For those reporting “3 ≥ months,” the exact number of months spent trying was used as their TTP. For women who reported their cycle length, TTP was adjusted to reflect the number of cycles rather than months. For women who reported their cycle length, TTP was adjusted to reflect the number of cycles rather than months. To investigate infertility specifically, we classified couples as experiencing infertility if they had tried to conceive for 12 months or more before succeeding, based on the women’s reports (9). Couples who had conceived through ART were also excluded from this analysis. Couples who did not have a planned pregnancy, used contraceptives at conception, or conceived through ART were excluded from this analysis.

Information on the use of ART, the main and contributing reasons for using ART, and ART treatment modality was obtained through linkage with the Medical Birth Registry of Norway. As a sub-analysis of the main reason for ART use, women registered for having endometriosis, ovulatory disorders, or tubal factor infertility as the main reason for ART use were classified as “main female-factor infertility”. Men registered for having sperm factor infertility as the main reason for ART use were classified as “main male-factor infertility”. Similarly, as a sub-analysis of contributing reasons to ART use, women registered for having endometriosis, ovulatory disorders, tubal factor infertility or uterus anomalies as contributing reasons for ART use were classified as “contributing female-factor infertility”. Similarly, men registered for having sperm factor infertility as a contributing reason for ART use were classified as “contributing male-factor infertility”.

### Statistical analyses

To examine the effect of TL on fecundability, infertility, and the use of ART, we examined the three measures of TL separately, *i.e.*, (i) aTL measured by Southern Blot, (ii) GRS for TL, and (iii) genetically predicted aTL. For the analysis of fecundability, we further used proportional probability regression and a discrete survival approach to estimate fecundability ratios (FRs), using the various TL measures as exposures and menstrual cycles as the unit of time in each analysis. This allowed us to estimate the relative difference in the probability of conceiving within a given menstrual cycle according to increasing levels of the three TL measures. This approach assumes that there is no disproportionate effect of any variables on the probability to conceive within a specific cycle. We censored the TTP at twelve cycles as this is when couples may seek infertility treatment. We also investigated the possibility of non-linear associations between the various TL measures and fecundability using generalized additive models (GAMs) with restricted cubic splines using the *mgcv* R package. We assessed model fit based on the effective degrees of freedom (EDF) and the Akaike information criterion (AIC) (33). For the analyses of differences in propensity to infertility and ART use according to increasing levels of the three TL measures, we used logistic regression to estimate the odds ratio (OR) for each of these outcomes.

The analyses of aTL were further adjusted for age (continuous), pre-pregnancy body mass index (BMI; continuous; kg/m^2^), highest completed or ongoing education level (categorical; university and high school or below), and smoking status (categorical; non-smoker, former smoker, and smoker during the last three months before pregnancy). In the analyses using GRS for TL and genetically predicted TL as exposures, we adjusted for age (continuous) and the first five genomic principal components (continuous). When analysing GRS for TL, we used age at birth for the MoBa index pregnancy as an alternative to the age when TL was measured. To ensure that these measures are comparable, we investigated the associations with fecundability, infertility, and ART use per one standard deviation (SD) increase in the TL measures.

### Sensitivity analyses

Since TL was only measured in women aged 30 years or above and their partners in the ART group, we conducted a sensitivity analysis excluding women and men below the age of 30 from the non-ART comparison (the reference group). Given that a couple’s TL can be correlated due to assortative mating, we carried out another sensitivity analysis where we mutually adjusted for the TL measures of partners.

## Results

Our study population included 1,054 women and 965 men in the analysis of aTL. Overall, 24,645 women and 18,339 men were included in the analysis of GRS for TL, and 958 women and 920 men in the analysis of genetically predicted TL (Fig. 1). Among those assessed for aTL and genetically predicted TL, 15% of couples had spent more than a year trying to conceive, whereas approximately 20% of couples had used ART to conceive. The mean age was 32 years (standard deviation [SD] = 4 years) for women and 34 years (SD = 5 years) for men. Within the subsample of the study population with data on GRS for TL, 11% of couples had spent more than a year trying to conceive, whereas 4% of couples had used ART to conceive. The mean age within this sample was 29 years (SD = 4 years) for women and 32 (SD = 5) for men.

Across all groups, those with infertility who conceived through sexual intercourse or who conceived through ART typically had a slightly higher BMI but were otherwise similar in terms of education level and smoking behaviour compared to those who spent less than a year to conceive (Table 1, Supplementary Table S1). The Pearson correlation coefficient for aTL between partners was 0.31 (Supplementary Figure S3). The GRSs were associated with longer aTL in both sexes, explaining 6% of aTL variation (Supplementary Figure S5). Using the first-stage F-statistic to test the strength of the association between GRS and TL measured by Southern blot, we found no signs of weak instrument bias (F-statistic > 10, Supplementary Figure S5; Supplementary Table S2).

### Fecundability and infertility

**Figure 2:**
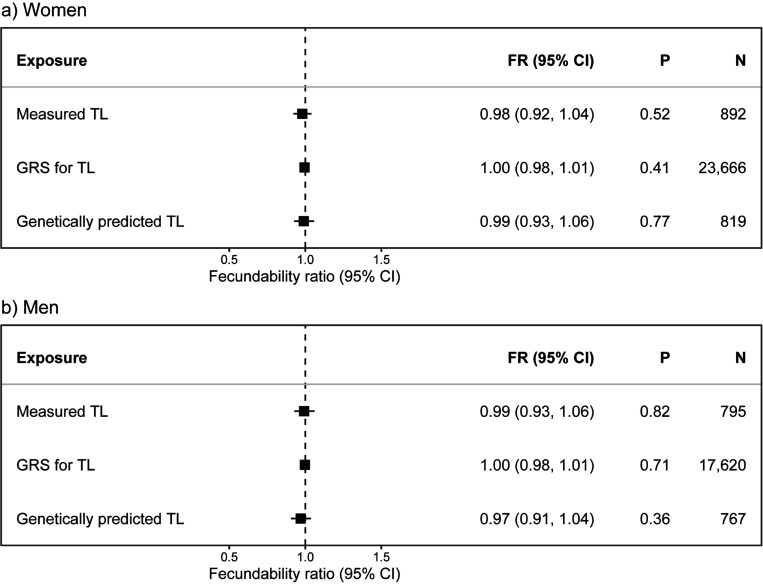
The associations between telomere length and fecundability. The associations between one standard deviation (SD) increase in age-adjusted telomere length (aTL) measures and fecundability in a) women and b) men.

**Figure 3:**
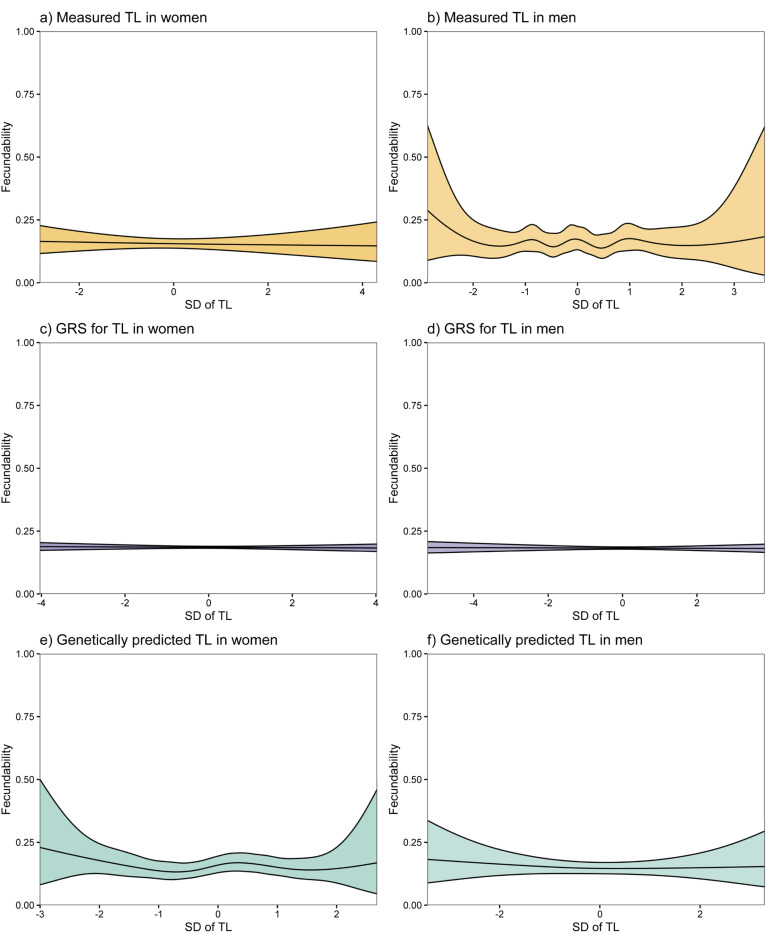
Non-linear associations between telomere length and fecundability. The non-linear associations between one standard deviation (SD) in age-adjusted telomere length (aTL) for a) women and b) men, genetic risk scores (GRSs) for TL for c) women and d) men, and genetically predicted aTL for e) women and f) men, in relation to fecundability. The colour scheme is the same as in the flow chart in Figure 1.

**Figure 4:**
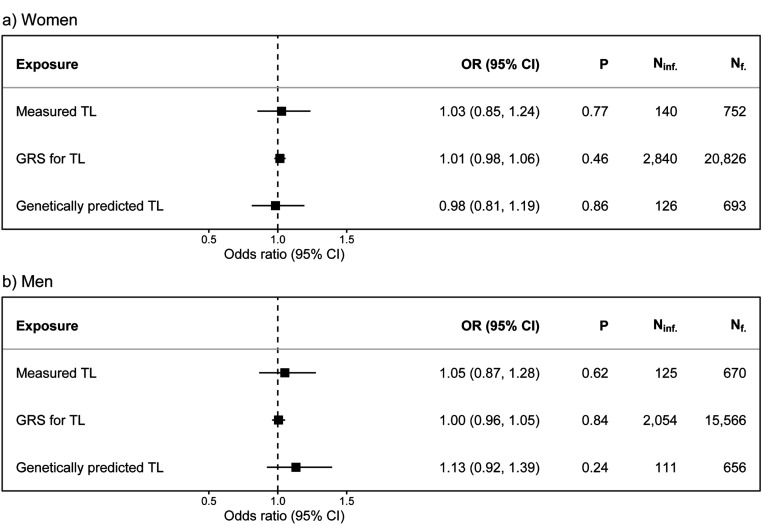
The associations between telomere length and infertility. The associations between one standard deviation (SD) increase in age-adjusted telomere length (aTL) measures and infertility for a) women and b) men.

We found no significant associations between any of the TL measures and fecundability in women or men (Fig. 2). The effects observed were mostly proportionate across menstrual cycles (Supplementary Figure S6 and S7). Furthermore, there was no strong evidence of any non-linear relationships between aTL and fecundability across the different TL measures (Fig. 3; Supplementary Table S3). Similarly, we found no significant associations between any of the TL measures and infertility in either sex (Fig. 4).

### ART

**Figure 5:**
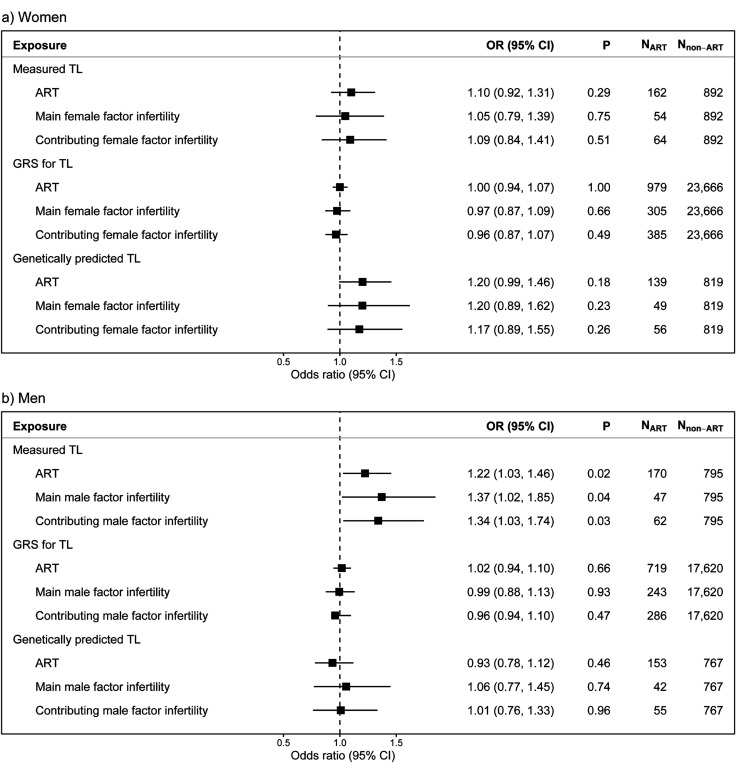
The associations between telomere length and use of assisted reproductive technologies. The association between a standard deviation (SD) increase in age-adjusted telomere length (aTL) measures and having conceived through assisted reproductive technologies (ART) in a) women and b) men.

We found no significant associations between any of the TL measures and use of ART in women (Fig. 5).In men, however, we found an association between longer aTL and increased risk of ART use (OR, 1.22; 95% confidence interval [CI], 1.03–1.1.46). The latter result was consistent when investigating both male factor infertility as the main reason for using ART (OR, 1.37; CI, 1.02–1.85) and as any reason for using ART (OR, 1,34; CI, 1.03–1.74). However, the increased risk of using ART with longer TL among men was not observed when investigating GRS for TL and genetically predicted aTL.

### Sensitivity analyses

The results were consistent when we limited our study population to include only women aged 30 years or above and their partners for the analyses related to ART use (Supplementary Figure S8) and when adjusting for partners’ aTL in all analyses (Supplementary Figure S9, S10 and S11).

## Discussion

To our knowledge, this is the first study to investigate the relationship between TL, fecundability, and ART use. We found no significant associations between TL measures and fecundability or infertility in this large population-based study. However, in men, we found a higher likelihood of ART use among men with longer aTL, a pattern not found in women. Importantly, this increased likelihood of ART use in men with longer telomeres was not replicated when we used GRS for TL or genetically predicted aTL, suggesting the possibility of unmeasured confounding factors or bias influencing this outcome (34).

Our findings contrast with those of earlier studies reporting an association between shorter TL and infertility-related phenotypes in women, such as PCOS and endometriosis, as well as association between shorter TL and sperm quality in men (15, 16, 21). However, the fact that many studies in the literature find conflicting results may indicate that there is no true relationship between TL and infertility, which is in line with our main finding. Any potential association between TL and infertility in men needs to be validated.

Differences in TL measurement techniques may partly account for the inconsistent findings across studies. These methods range from polymerase chain reaction (qPCR)-based approaches to various fluorescent *in situ* hybridization (FISH) methods and Southern blotting (35–38). Additionally, telomere dynamics vary across different tissues, especially between germ cells and somatic cells (39). TL can be measured in different tissues and cells, including stromal cells, leukocytes, endometrial cells, tubal epithelial cells, granulosa cells or oocytes, and sperm cells (15). While telomeres shorten with age in most tissues, they remain stable in some, like testis and ovaries (40). Moreover, while there is considerable evidence linking oxidative stress and TL shortening *in vitro,* the impact of oxidative stress on TL shortening *in vivo* is less understood (41). The relatively small sample sizes in selected populations used in some studies may also lead to noise results. These methodological shortcomings could significantly affect the interpretation of the effect of TL on infertility-related phenotypes.

The observed modest association between longer TL and a higher likelihood of ART use in men remained stable, both when male factor infertility was considered as a main reason, when it was considered as a contributing reason for ART use, and when mutually adjusting for their partners’ aTL. However, this association was not replicated in analyses involving GRS for TL or genetically predicted aTL, indicating that the relationship might not be causal. Our results may also be biased by selection as men using ART was the smallest subsample in our study. This could induce associations of similar magnitude, indicating that the observed association could instead be influenced by lifestyle factors linked to TL, such as nutrition or smoking (42). Indeed, smoking is known to be an important external source of reactive oxygen species, which are predicted to negatively affect the triple guanine residues in telomeric repeats (43, 44).

Several studies have identified a positive correlation between offspring’s leukocyte TL and paternal age at conception, even after adjusting for offspring’s age (45–48). To examine whether potential effects of paternal age at conception (PAC) could explain the significant association between aTL and ART use in men, we calculated paternal residual aTL by regressing aTL on fathers’ age at birth when the women and men in our study population were born, and then repeating the analyses. The Pearson correlation between fathers’ ages at birth and aTL was 0.11 for women and 0.14 for men (Supplementary Figure S4). We found significant PAC effects, although adjusting for PAC in the analysis of aTL and ART use in men did not alter the results significantly, except for a slight strengthening of the association (Supplementary Figure S12, S13 and S14).

Studies have suggested that the TL in sperm cells increases with age, and it has been proposed that a more robust telomerase activity in these cells could be a mechanism for the observed PAC effect (48). Moreover, since older men have lower sperm counts, the amount of telomerase available per sperm cells is also greater in older men. Consequently, the offspring of older fathers tend to have longer telomeres in leukocyte cells (49). However, this significant PAC effect did not explain the association between longer telomeres and increased likelihood of using ART among men in our study. An alternative explanation might be related to common mechanistic patterns associated with male factor infertility. For instance, it has been proposed that male factor infertility, including low sperm count, may cluster within families (50, 51), potentially leading to longer telomeres in the sperm of fathers and the leukocytes of their offspring. This familial pattern could also contribute to the observed increased likelihood of ART use among men from such families. Further studies, with detailed analysis of male factor infertility across generations, are needed to elucidate the link between longer TL and the higher likelihood of ART use in men.

Key strengths of our study include our ability to investigate the associations using three measures of TL: aTL assessed through direct measurements by Southern blot, GRS constructed for TL from GWAS summary statistics, and genetically predicted aTL. Our measurements of TL were obtained using Southern Blot, which ensures higher accuracy compared to, for example, PCR-based methods. We also had detailed information on TTP, allowing us to estimate FRs. Moreover, we were able to investigate the risk of infertility among couples who conceived through sexual intercourse and those who conceived through ART. We also had detailed information on the reasons for using ART.

The limitations of our study include the fact that MoBa is a pregnancy cohort. Since all the participants were recruited based on having achieved a pregnancy, we were unable to investigate associations in women and men the most severe phenotypes, such as those who never conceived or experienced an early pregnancy loss. Given the lack of data on childless women and men, we were also unable to investigate the probability of using ART independent of treatment outcome. Recall bias may also influence our findings, given potential inaccuracies in participants’ recollections of their TTP. Lastly, MoBa comprises a selected and homogeneous group in general, representing women and men in a higher socioeconomic bracket compared to the population at large. This homogeneity may have contributed to TL being a poor biomarker of health-related factors in MoBa specifically (52).

## Conclusion

In conclusion, we found no significant evidence that aTL influences fecundability, infertility, or use of ART in either women or men. An exception was a modest association between longer measured telomeres and an increased likelihood of ART use in men. However, this association was not observed when examining the same relationships using GRSs for TL or genetically predicted aTL, underscoring the need for validation in other cohorts. Overall, our results suggest that TL may not serve as a robust and reliable biomarker for assessing fecundability, infertility, or ART use in the MoBa cohort, a finding that needs to be validated in other cohorts with access to similar data.

## Figures and Tables

**Table 1:** Characteristics of the study population.

	Women			Men		
	Fertile	Infertile	ART use	Fertile	Infertile	ART use
N (%)	784	153	173	753	142	179
Age, median (IQR)	32.1 (28.6, 33.8)	33.3 (31.7, 34.8)	34.1 (32.4, 35.9)	32.7 (29.9, 35.8)	34.0 (31.1, 37.8)	35.8 (33.6, 38.8)
TL, median (IQR)	7.8 (7.3, 8.3)	7.7 (7.3, 8.1)	7.9 (7.3, 8.3)	7.7 (7.2, 8.1)	7.7 (7.3, 8.1)	7.8 (7.3, 8.3)
TTP, median (IQR)	2 (2, 5)	17 (12, 26)		3 (2, 5)	17 (12, 28)	
BMI, median (IQR)	22.8 (21.1, 25.3)	23.7 (21.3, 27.5)	22.9 (21.4, 25.1)	25.4 (23.5, 27.7)	25.7 (24.0, 27.6)	25.6 (24.3, 27.8)
BMI, N (%)	770 (98.2)	153 (100.0)	171 (98.8)	740 (98.3)	139 (97.9)	177 (98.9)
Missing, N (%)	14 (1.8)	0 (0.0)	2 (1.2)	13 (1.7)	3 (2.1)	2 (1.1)
Higher education, N (%)	579 (73.9)	110 (71.9)	148 (85.6)	455 (60.4)	85 (59.6)	120 (67.0)
Lower education, N (%)	187 (23.9)	39 (25.5)	17 (9.8)	282 (37.9)	57 (40.1)	57 (31.9)
Missing, N (%)	18 (2.3)	4(2.6)	8 (4.6)	13 (1.7)	0 (0.0)	2 (1.1)
Non-smoker, N (%)	406 (51.8)	78 (51.0)	90 (52.0)	297 (39.4)	55 (38.7)	66 (36.9)
Smoker > 3 mo. ago, N (%)	158 (20.2)	29 (19.0)	45 (26.0)	192 (25.5)	36 (25.4)	59 (33.0)
Smoker last 3 mo., N (%)	212 (27.0)	45 (29.4)	36 (20.8)	192 (25.5)	45 (31.7)	48 (26.8)
Missing, N (%)	8 (1.0)	1 (0.6)	2 (1.2)	72 (9.6)	6 (4.2)	6 (3.4)

Characteristics of the participants in the study population with measurements of telomere length.

## Data Availability

Data from the Norwegian Mother, Father and Child Cohort Study and the Medical Birth Registry of Norway used in this study are managed by the national health register holders in Norway (Norwegian Institute of public health) and can be made available to researchers, provided approval from the Regional Committees for Medical and Health Research Ethics (REC), compliance with the EU General Data Protection Regulation (GDPR) and approval from the data owners. The consent given by the participants does not open for storage of data on an individual level in repositories or journals. Researchers who want access to data sets for replication should apply through helsedata.no. Access to data sets requires approval from The Regional Committee for Medical and Health Research Ethics in Norway and an agreement with MoBa.
